# Influence of Sex of Stranger on Responses of Shelter Dogs during Canine Behavioral Evaluations

**DOI:** 10.3390/ani13152461

**Published:** 2023-07-30

**Authors:** Betty McGuire, Andrew Song

**Affiliations:** 1 Department of Ecology and Evolutionary Biology, Cornell University, Ithaca, NY 14853, USA; 2 Department of Information Science, Cornell University, Ithaca, NY 14853, USA; ajs557@cornell.edu

**Keywords:** aggression, familiarity, risk factor, temperament test, undersocialized dogs

## Abstract

**Simple Summary:**

Dogs are often less comfortable around unfamiliar men than unfamiliar women, yet most studies of stranger-directed aggression have not examined whether the sex of the stranger is a risk factor. We analyzed data collected by staff at a NY shelter to determine whether dogs responded differently to unfamiliar men and unfamiliar women during the Stranger test of the behavioral evaluation. Of the 283 dogs tested, 26 were undersocialized and from the same home; 19.2% were assessed as showing no concerning behavior during the Stranger test. Of the 257 remaining dogs, 89.9% were assessed as showing no concerning behavior. Dogs tested with a male stranger (*n* = 55) had significantly higher scores on the test, indicating greater uneasiness, than dogs tested with a female stranger (*n* = 202). However, the mean score for dogs tested with a male stranger (2.2) fell within the range of scores considered not concerning by the shelter (1–3). In summary, we found that the sex of a stranger influenced dog responses during the Stranger test, but in practice, our findings do not indicate that changes are needed in how shelters conduct or interpret tests for stranger-directed aggression. Our findings also underscore the importance of socialization for dogs.

**Abstract:**

In many situations, domestic dogs display greater uneasiness with unfamiliar men than unfamiliar women. However, little is known about whether the sex of an unfamiliar person is a risk factor for stranger-directed aggression, especially with respect to behaviors less intense than biting. We analyzed data collected by behavioral staff over a 27-month period (*n* = 283 dogs) at a New York shelter to determine whether the sex of an unfamiliar person influenced behaviors assessed during the Stranger test of the canine behavioral evaluation. Scores ranged from 1 (calm and friendly) to 5 (will not approach stranger or unsafe to allow an approach). No concerning behaviors (scores 1–3) were assessed for 19.2% of 26 undersocialized dogs from one home and 89.9% of the remaining 257 dogs. Within the group of 257, those tested with a male stranger had significantly higher scores than those tested with a female stranger; the effect size was small to moderate. Thus, we found that dogs responded differently to male and female strangers during this testing situation, but from a practical standpoint, our findings do not warrant adjustments in how shelters conduct or interpret tests for stranger-directed aggression. Our findings also highlight the importance of early exposure to different people and situations for dogs.

## 1. Introduction

Domestic dogs—in shelters and other environments—respond differently to unfamiliar men and unfamiliar women, with the general pattern being greater uneasiness with unfamiliar men, sometimes more pronounced in male dogs than female dogs. Shelter dogs enrolled in an enhanced human interaction program showed improved sociability toward unfamiliar women but not unfamiliar men [1]. At another shelter, when an unfamiliar person stood in front of their cage, dogs watched and barked longer when the person was a man rather than a woman [2]. The sex of an unfamiliar walker influenced scent-marking behavior during leash walks of mature shelter dogs: when walked by an unfamiliar man, male dogs urinated less frequently, were more likely to revert to the juvenile urinary posture (no hindlimb raised), and were less likely to defecate than when walked by an unfamiliar woman [3]. The sex of an unfamiliar walker did not influence frequency of urination or urinary posture in female dogs; however, like male dogs, female dogs were less likely to defecate when walked by an unfamiliar man than an unfamiliar woman. At a different shelter, dogs displayed behaviors associated with vigilance (gazing at the handler) and stress (lip-licking) more frequently when walked by men than women and spent less time with their tail in the high position when walked by men than by women [4]. Dogs living in other environments show similar responses to unfamiliar men; those enrolled in a training program for guide dogs made less frequent physical contact with unfamiliar men than unfamiliar women [5]. Finally, in a commercial kennel environment, male dogs made less frequent direct physical contact with and spent less time near an unfamiliar man than an unfamiliar woman; in contrast, female dogs did not differ in their behavior toward an unfamiliar man and an unfamiliar woman [6].

In the same way, another dog-human interaction that has been examined with respect to familiarity is aggression directed by dogs toward people; in most studies, aggression directed at strangers is considered separately from aggression directed at household members. Stranger-directed aggression is commonly assessed in animal shelters that conduct canine behavioral evaluations before making dogs available for adoption; about 6–8% of dogs were assessed as showing concerning or dangerous behaviors in the presence of strangers [7,8]. Aggression toward unfamiliar people has also been studied in dogs living in homes. Prevalence measures based on owner-completed questionnaires vary widely, perhaps reflecting different scoring systems and dog populations (5–10%, 9–11; 24–26%, 12,13; 78%, 14). Research has focused also on identifying risk factors for stranger-directed aggression, including characteristics of the dog, the owner, and the home environment [9,10,11,12,13,14,15,16,17,18]. However, with the exception of literature on victim risk factors for dog bites, which typically reports a predominance of male victims [19,20,21,22], we could find no information on whether the sex of an unfamiliar person influences other dog behaviors associated with stranger-directed aggression, such as barking, raising hackles, and growling. Sex of the stranger was not reported in previous studies of shelter dogs [7,8] or included in those using owner-completed questionnaires, which asked how dogs respond to a “stranger” or an “unfamiliar person,” without specifying sex [9,10,11,12,13,14,15,16,17,18].

In the present study, we examined whether dogs at a New York animal shelter reacted differently to an unfamiliar man or an unfamiliar woman during the Stranger test of the canine behavioral evaluation. During the Stranger test at this shelter, dogs are assessed as showing responses to the stranger ranging from calm and friendly to not being safe enough to allow the dog to approach the stranger. Based on findings from previous studies of dog reactions to unfamiliar men and unfamiliar women [1,2,3,4,5,6], we predicted that dogs tested with an unfamiliar man would display greater uneasiness than dogs tested with an unfamiliar woman. Given that studies have found uneasiness toward men to be more pronounced in male dogs than female dogs [3,6], whereas others have not found this pattern [1,2,4,5], we considered it equally likely for us to find either more pronounced uneasiness toward male strangers in male dogs or similar levels of uneasiness with male strangers in male and female dogs. In addition to providing information on an understudied risk factor for stranger-directed aggression generally, our findings may inform how shelters conduct and interpret tests with strangers. These shelter-related applications are important because results from behavioral evaluations influence the development of in-shelter handling plans (e.g., whether a dog has a limited team of volunteers who must be introduced by staff) and special adoption efforts to make the best possible match between dog and adopter (e.g., whether a potential adopter’s household has frequent visitors).

## 2. Materials and Methods

This research was conducted under protocol 2012-0150, which was reapproved on 16 September 2021 by the Institutional Animal Care and Use Committee of Cornell University.

### 2.1. Study Shelter and Canine Records

We obtained canine records from the Tompkins County SPCA in Ithaca, NY, USA. The shelter is no-kill, open-admission, and uses scheduled intake and a conversation-based approach to adoptions. The records, entered by shelter staff into the PetPoint data management system, included behavioral results from the Stranger test of the shelter’s canine behavioral evaluation (Section 2.3) as well as intake data, from which we retrieved demographic information for each dog evaluated (sex, reproductive status, and age). Our study began 1 January 2021 (the time when, upon our request, the name of the stranger began being routinely recorded by staff for each test) and ended 2 April 2023. This time period yielded records for 283 dogs, which represented all dogs behaviorally evaluated. Some dogs were admitted to the shelter but not behaviorally evaluated, and therefore, were not part of our sample; these dogs included those retrieved by owners shortly after their arrival at the shelter (behavioral evaluations typically occur 3 days after intake), those euthanized at their owner’s request, and very rare instances in which dogs were identified at intake as unsafe to make available for adoption and euthanized. Behavioral evaluations might also be skipped for extremely undersocialized dogs, such as those not used to living inside a home and having routine contact with people; however, this, too, was a very rare occurrence (*n* = 1 dog during our study).

### 2.2. Dogs, Care, and Housing

The 283 dogs included juveniles (*n* = 55; from 4 months to <1 year), adults (*n* = 195; from 1 year to <8 years), and seniors (*n* = 33; ≥8 years); we did not include puppies because their behavioral evaluations differed from those of older dogs and their results were not entered into PetPoint. Included in the 283 dogs were 26 Chihuahuas or Chihuahua mixes (14 males and 12 females) from the same home. These dogs had very little exposure to people other than the owner (hereafter, described as undersocialized dogs). Most had been spayed or neutered before admission to the shelter (88.5%; 23/26) and all but one were either adults (57.7%; 15/26) or seniors (38.5%; 10/26). Given their unusual living situation before admission to the shelter, we excluded them from formal data analyses (Section 2.4). Chihuahuas and Chihuahua mixes from other homes were not excluded. Most dogs were mixed breeds; the number of purebred dogs in our study population was unknown (DNA testing was not performed).

We describe the housing and care of dogs from intake to behavioral testing, which is the relevant time period for the present study. Intake occurred in the Rescue building, where veterinary staff examined dogs, weighed them, and checked their teeth to estimate age. The intake exam also included vaccinations, flea control, a fecal exam, deworming, and a heartworm test. Following the intake exam, dogs were housed in the Rescue building in chain-link cages (indoor space, 2.2 m^2^, and outdoor run, 3.5 m^2^). Almost all dogs were individually housed; exceptions were made for dogs surrendered from the same household when staff judged the dogs should remain together (e.g., the undersocialized dogs were typically housed in pairs). Each cage contained a raised bed, blanket, water bowl, and toys; staff fed dogs between 08:00 and 09:00 h and between 14:00 and 15:00 h. Several times a day, staff either walked dogs or brought them to an outdoor enclosure. Typically, three days after intake, dogs were behaviorally evaluated and subsequently housed in the Pet Adoption Center, adjacent to the Rescue building.

### 2.3. Behavioral Evaluation

The canine behavioral evaluation at our study shelter is based on Sternberg’s Assess-a-Pet [23], subsequently modified by Bollen and Horowitz [24]. Present at each test were an evaluator from the shelter’s Behavior Program and a scribe, also a member of shelter staff. The evaluator was female, and three other female staff members rotated through the role of scribe, with one exception when a different female staff member (an animal control officer) served as scribe. The same female evaluator conducted all behavioral evaluations except two during our study period (*n* = 281 of 283 dogs); two of the female staff members typically serving as scribes conducted the other two evaluations. The evaluation includes nine tests, some of which have subtests. The first test, Cage presentation, takes place in the Rescue building, and then the dog is leashed and brought to a conference room in the Pet Adoption Center, where all subsequent tests are conducted. In Appendix A, we provide brief descriptions of all tests and subtests that are not the focus of the present study; below is a more detailed description of the Stranger test.

The Stranger test is the eighth test in the nine-test sequence of the canine behavioral evaluation (Appendix A). The stranger is at least 18 years old, unfamiliar to the dog, and may be either a male or female staff member or dog volunteer, wearing ordinary clothing (not scrubs). With the evaluator holding the dog’s leash, the test begins when the stranger knocks on the door of the conference room and the evaluator says, “Come in!” The stranger enters the room, stops at least 2 m away from the dog, and stares at the dog for 3 s. Next, the stranger takes a step forward, stops, and reaches toward the dog. Finally, the stranger squats down, facing sideways, and speaks to the dog in a friendly voice. The dog’s behavioral response to the stranger is scored as follows:

1. Remains calm and is friendly upon solicitation;
2. Is nervous about the stranger (ears back, tail tucked), but is friendly upon solicitation;
3. Alarm barks or growls and backs off completely, but is friendly upon solicitation;
4. Alarm barks, hackles up, growls, does not calm readily, but eventually is friendly in a cautious way upon solicitation;
5. Alarm barks, hackles up, growls, cannot settle, and either will not approach upon solicitation or is not safe to allow an approach.

The scribe writes her score on the evaluation form, and the evaluator reviews the score with reference to her own score. Any difference in score is discussed, and an agreement is reached regarding the score entered into PetPoint. Note that a particular score does not mean that a dog showed every behavior listed for that score. For example, dogs receiving a score of 5 may not alarm bark, raise hackles, or growl but simply avoid any interaction with the stranger; this information is recorded in the notes section of PetPoint. In the shelter scoring system, scores 1–3 are classified as “no concerning behavior”, a score of 4 is considered “concerning behavior”, and a score of 5 is “dangerous behavior”. The behavioral responses of dogs that receive a score of 5 and will not approach the stranger are considered dangerous because of the potential for defensive aggression should someone attempt to force interaction despite a dog’s attempts to get away or warn them away. Responses of dogs that receive a score of 5 and are assessed as unsafe to allow them to approach the stranger are considered dangerous because of the potential for offensive aggression; this assessment of unsafe to allow an approach is rare at our study shelter (e.g., of the 283 dogs in our sample, only one was considered unsafe to allow an approach; Section 3.1.1).

### 2.4. Data and Statistical Analyses

From each PetPoint record of the Stranger test, we retrieved the dog’s name, identification number, score (1–5), date of test, evaluator’s name, and all notes about the test, which included the name of the person serving as the stranger. From the name of the stranger, we assigned the sex of the stranger. BM has conducted research at this shelter for over 10 years, so she is very familiar with both dog volunteers and staff members, making the assignment of the sex of a stranger based on the recorded name straightforward. We checked paper copies of the evaluations to retrieve the name of the scribe for each test. Dogs that are adopted and returned to the shelter are behaviorally evaluated again; for dogs with two or more behavioral evaluations during our study period (*n* = 23), we used results from their first evaluation to make testing conditions across dogs as similar as possible.

For the 26 undersocialized dogs, we simply report descriptive information about their performance on the Stranger test; two were evaluated by staff members other than the main evaluator. Having excluded these 26 undersocialized dogs, we were left with 257 dogs for data analyses; all were assessed by the same evaluator.

Of the 257 tests, 55 (21.4%) involved a male stranger and 202 (78.6%) involved a female stranger; the much larger number of female strangers reflects the female bias in both dog volunteers and staff members (in most positions) at our study shelter [3]. Table 1 shows the demographic characteristics of the 257 dogs tested with either a male or female stranger. We used chi-square tests to evaluate whether dogs tested with a male stranger had generally similar demographic characteristics as those tested with a female stranger; we found no significant differences (sex: *X*^2^ = 0.05, *d.f.* = 1, *p* = 0.82; reproductive status: *X*^2^ = 0.66, *d.f.* = 1, *p* = 0.42; age class: *X*^2^ = 1.37, *d.f.* = 2, *p* = 0.51).

We calculated the prevalence of concerning or dangerous behavior for the Stranger test, defined as the number of dogs assessed as showing either concerning or dangerous behavior/number of dogs tested. Separate prevalence measures are provided for the 26 undersocialized dogs and the 257 dogs. We used a standard least squares model to examine whether the sex of the stranger, along with the demographic characteristics of the 257 dogs—sex, reproductive status, and age class—predicted a dog’s score on the Stranger test. We examined the main effects and the one two-way interaction we were interested in, sex of dog by sex of stranger. This interaction was not significant, so we dropped it from the final model (*F* = 1.89, *d.f.* = 1, *p* = 0.17). We used Student’s *t*-tests when comparing means for both sex and reproductive status and Tukey’s HSD to correct for multiple comparisons when comparing means for age class. Because of sample size differences, especially with regard to the number of dogs tested with either a male stranger or a female stranger, we used Hedges’ *g* to calculate effect sizes. We set the *p* value threshold at *p* < 0.05 and used JMP Pro (version 15.0.0) for all statistical analyses except the calculation of effect sizes, which was performed using Social Sciences Statistics (https://www.socscistatistics.com (accessed on 28 May 2023).

## 3. Results

### 3.1. Main Study with 257 Dogs

#### 3.1.1. Prevalence

The vast majority of dogs (89.9%; 231/257) were assessed as showing no concerning behavior during the Stranger test. The prevalence of concerning behavior was 6.6% (17/257), and the prevalence of dangerous behavior was 3.5% (9/257). During our study period, only one dog was assessed as unsafe to allow him to approach the stranger, but this was during his second behavioral evaluation, and we only used results from first evaluations. He was tested upon first entering the shelter (score of 3 on the Stranger test), adopted and returned a few months later, and evaluated again (score of 5, unsafe to allow an approach). Nevertheless, he was successfully adopted after being on an in-shelter handling plan for 1 month (limited team of volunteers who must be introduced by staff). Thus, for the first behavioral evaluations, none of the dogs with a score of 5 was assessed as unsafe to allow them to approach the stranger; all would not approach upon solicitation.

Of the 26 dogs with a score of either 4 or 5, only one was described as trembling and frozen in place; this dog did not bark, raise hackles, or growl. The other 25 dogs with scores of 4 or 5 displayed at least one of these three behaviors.

#### 3.1.2. Response to a Stranger in Relation to the Sex of the Stranger and Demographic Characteristics of Dogs

Descriptive statistics for scores on the Stranger test in relation to the sex of the stranger and dog demographic characteristics are shown in Table 2. Dogs tested with a male stranger had significantly higher scores on the Stranger test than dogs tested with a female stranger, indicating greater uneasiness with male strangers (*F* = 6.81, *d.f.* = 1, *p* < 0.01; Table 2). The effect size was in the small to moderate range (Hedges’ *g* = 0.36).

Reproductive status significantly influenced scores on the Stranger test, with intact dogs having higher scores than spayed/neutered dogs (*F* = 5.34, *d.f.* = 1, *p* < 0.03; Table 2); the effect size was small (*g* = 0.29). Sex and age class of dogs did not influence scores on the Stranger test (sex: *F* = 0.18, *d.f.* = 1, *p* = 0.67; age class: *F* = 1.98, *d.f.* = 2, *p* = 0.14; Table 2).

### 3.2. Undersocialized Dogs

Scores on the Stranger test for the 26 undersocialized dogs from one household were as follows: 1 (*n* = 1); 2 (*n* = 4); 3 (*n* = 0); 4 (*n* = 5); and 5 (*n* = 16). Thus, 19.2% (5/26) of these dogs were assessed as showing no concerning behavior (scores 1–3), 19.2% as showing concerning behavior (score of 4), and 61.6% as showing dangerous behavior (score of 5). None of the dogs with a score of 5 was assessed as unsafe to allow them to approach the stranger; all would not approach the stranger upon solicitation. For all 21 dogs with a score of either 4 or 5, notes in the PetPoint files described them as showing the following responses: trembling, hiding, freezing, and shutting down. Notes further indicated these dogs did not engage in alarm barking, raising hackles, or growling. When considering all 26 undersocialized dogs, the mean score (±*SD*) for those tested with a male stranger was 4.83 ± 0.41 (*n* = 6) and for those tested with a female stranger, 4.00 ± 1.38 (*n* = 20).

All of the undersocialized dogs were released for adoption. All 26 were adopted, and three were returned (11.5%): one for biting when the adopter tried to remove him from a hiding place, one for warning behaviors but no biting, and the other after a year, when the adopter could no longer care for her (this dog was the only one of the 26 to have a score of 1 on the Stranger test). All three dogs were adopted again and not returned to the shelter.

## 4. Discussion

The vast majority of the 257 dogs behaviorally evaluated during our study period (2021–2023) were assessed as showing no concerning behavior on the Stranger test (89.9%), with the prevalence of concerning or dangerous behavior at 10.1% (6.6% concerning + 3.5% dangerous). A prevalence of 10.1% is slightly higher than the 5.8% previously reported for dogs tested at this shelter from 2014 to 2019 [7]. This likely reflects that the current study included all dogs tested, whereas McGuire et al. [7] were studying length of stay at the shelter, so they only included dogs that were tested, released for adoption, and housed at the shelter until adoption (i.e., not in foster homes; typically, dogs placed in foster homes are either especially fearful or have medical conditions such that a home environment is more suitable than the shelter environment). Thus, in contrast to McGuire et al. [7], our study population included dogs that were tested and either returned to their owner, transferred to a rescue, or placed in a foster home; some of the dogs transferred to a rescue or placed in a foster home likely had more intense responses during the behavioral evaluation, possibly towards strangers, than those tested, released for adoption, and housed at the shelter. Our prevalence measure of 10.1% is generally similar to those from another shelter [8] and some studies using owner-completed questionnaires [9,10,11]. Other studies based on owner-completed questionnaires reported higher prevalence measures for stranger-directed aggression [12,13,14].

Dogs tested with a male stranger had higher scores on the Stranger test, which indicates greater uneasiness, than dogs tested with a female stranger. Although this difference was statistically significant, the effect size was in the small to moderate range, and both means (male stranger, 2.22, and female stranger, 1.81) fell within the range of scores considered no concerning behavior on the test (1–3). We did not find a significant interaction between the sex of the dog and the sex of the stranger, indicating that male and female dogs responded in a similar manner to male strangers. Our findings from the Stranger test are consistent with results from other studies indicating greater uneasiness with unfamiliar men than unfamiliar women in both male and female shelter dogs [1,2,4] and dogs in a guide dog training program [5]. In an earlier study at the Tompkins County SPCA, both male and female dogs were less likely to defecate when walked by an unfamiliar man than an unfamiliar woman, again indicating similar levels of uneasiness with male strangers [3]. However, male but not female dogs displayed lower rates of urination and were more likely to revert to the juvenile urinary posture when walked by an unfamiliar man than by an unfamiliar woman [3]. The sex of the dog seems to modulate the response differently based on the specific behavior under study.

In regard to the greater uneasiness shown by dogs toward unfamiliar men than unfamiliar women in our study, it is possible that—from a dog’s perspective—unfamiliarity with men has two components. First, male staff members and male dog volunteers serving as strangers are unknown to the dogs as individuals, thus “unfamiliar”. Second, given the female bias in staff and dog volunteers at our study shelter [3], male strangers are also members of a less familiar group, human males. The possibility that unfamiliarity could have two components might suggest that shelters make efforts to recruit more males when filling staff and volunteer positions; this could ease the first interactions between dogs and potential adopters who are male.

Of the three demographic characteristics examined, only reproductive status was a significant predictor of score on the Stranger test, with scores of intact dogs higher than those of neutered and spayed dogs. However, the effect size was small, and the means were within the range considered to be no concerning behavior (intact, 2.06; neutered/spayed, 1.73). In an earlier study based on nearly 5 years of data at our study shelter, reproductive status was not significantly associated with the likelihood of concerning or dangerous behavior on the Stranger test [25]. Studies using owner-completed questionnaires reported no effect of reproductive status on stranger-directed aggression [15,18], and a study based on phone interviews found no decrease in stranger-directed aggression in male dogs following neutering [26]. Farhoody et al. [17] reported a significant increase in the odds of stranger-directed aggression in neutered or spayed dogs when compared to intact dogs, but this result was driven by dogs whose surgeries were performed between 7 and 12 months of age. The reasons for these divergent findings are unclear.

We found that the sex of the dog did not influence scores on the Stranger test. Our finding of no effect of dog sex on stranger-directed aggression is consistent with those based on owner-completed questionnaires [12,15,18]. In contrast, Takeuchi et al. [27] found that male dogs tended to be over-represented in cases of stranger-directed aggression at a behavior clinic. Finally, we found that the age of the dog did not influence scores on the Stranger test, which agrees with findings from one study based on an owner-completed questionnaire [18]. However, other studies have found either an increase in stranger-directed aggression with age [9,12,15] or mixed results, with adults more likely to be aggressive than adolescents and seniors [14]. Our present findings that neither sex nor age class influenced scores on the Stranger test are consistent with findings from earlier research at our study shelter [25].

The prevalence of concerning or dangerous behavior during the Stranger test for the 26 undersocialized dogs from one home was 80.8% (19.2% concerning + 61.6% dangerous). None of these dogs with concerning or dangerous behavior displayed alarm barking, raising hackles, or growling. Instead, they hid, trembled, and froze, and those with a score of 5 would not approach the stranger, leaving open the possibility for defensive aggression should their signals be ignored and interaction forced. The conditions of the Stranger test (e.g., an unfamiliar room in the shelter, two new people with whom they have only spent a few minutes, then a knock on the door, followed by a third person they have never met) seemed especially stressful for these dogs, on top of the already challenging conditions of shelter life (e.g., loud sounds, new smells, many unfamiliar people and dogs). The responses displayed by the undersocialized dogs in our study are typical of moderately to extremely fearful dogs in shelters, of which about one-third come from hoarding situations [28], the source of the dogs in our study. The high prevalence of fearful behaviors in these dogs highlights the importance of exposing dogs, especially when young, to different people and situations [29]. Despite their fearfulness in the shelter, all were adopted, and only three were returned (11.5%); this likelihood of return is similar to the 13.7% reported for small dogs at this shelter from 2014 to 2019 [30]. The three returned dogs were subsequently adopted and not returned to the shelter. Collins et al. [28] reported similar positive outcomes from a much larger sample of fearful dogs that went through a behavioral rehabilitation program: 99% of dogs that completed the program were adopted, with 88% of adopters very satisfied and 8% somewhat satisfied with their dog.

Our study has several limitations. First, canine behavioral evaluations at shelters have been criticized for their lack of rigorous scientific validation and poor predictability of many post-adoption behaviors [8,31,32,33]. However, proponents view these evaluations as one of several sources of information about dogs in shelters [24,34]. Second, although the Stranger test attempts to mimic a stranger knocking on the door of a dog’s home, the contexts and settings of the two situations are very different. Whereas dogs in their homes may be defending household members and/or their home area, dogs in shelters have typically been housed there for only a few days before testing and have interacted with the evaluator and scribe for only a few minutes before someone the dog has never met knocks on the conference room door. Additionally, the dogs are leashed during the Stranger test, whereas dogs are likely not leashed in their home when a stranger approaches. The fact that our measure of prevalence of concerning or dangerous behavior during the Stranger test (10.1% for the 257 dogs) is similar to measures found in some studies based on owner-completed questionnaires [9,10,11] suggests that perhaps the sound of knocking signals to dogs the arrival of a stranger, even in a setting very different from a home [25]. Third, shelters are challenging environments for dogs, and certain aspects of the Stranger test (e.g., the door opening following knocking and possible changes in leash tension) might induce fear or arousal in the dogs right before they see the stranger, potentially resulting in false positives. Fourth, because we did not videotape the Stranger tests, we could not assess intra-rater reliability, which would be useful given the same evaluator conducted all of the Stranger tests for the 257 dogs formally analyzed. Fifth, we did not determine the particular stimuli used by the dogs to discriminate the sex of the stranger. In the setting of the Stranger test, potential stimuli include visual (e.g., size and appearance of the stranger), olfactory (e.g., androgen-based cues), and auditory cues (e.g., voice of the stranger). Sixth, because our data came from a single shelter, our findings may not apply to other shelters with different dog populations and behavioral evaluations [35,36]. Finally, given the female bias in the two sources of strangers at our study shelter—dog volunteers and shelter staff [3]—our sample of dogs tested with a male stranger (*n* = 55) was much smaller than our sample tested with a female stranger (*n* = 202). Herzog [37] recently highlighted the large numbers of women in professions related to animal care and research on human-animal relationships; he also noted that studies of the human-animal bond often have too few male participants. Herzog [37] suggested authors make the gender breakdown of study participants clear, which we have done here for those administering the Stranger tests and serving as the stranger. We also checked the demographic characteristics of dogs tested with male strangers versus female strangers to make sure the two groups of dogs were similar in terms of sex, reproductive status, and age class.

The present study used a between-subjects experimental design. It would be interesting to examine dogs’ reactions to male and female strangers using a within-subject design. Given questions about whether Stranger tests with shelter dogs adequately mimic situations in homes, it would be best to conduct this research with dogs in their own homes. Such research might further contribute to our understanding of victim risk factors for canine aggression directed at strangers.

## 5. Conclusions

We found that dogs tested with an unfamiliar male had significantly higher scores than dogs tested with an unfamiliar female on the Stranger test of the canine behavioral evaluation; this finding provides another example of dogs responding differently to unfamiliar men and unfamiliar women. However, the effect size was small to moderate, and the mean score for dogs tested with a male stranger fell within the range of scores grouped as no concerning behavior on this particular test of the evaluation. From a practical standpoint, our findings do not indicate that changes are needed in how shelters conduct or interpret tests for stranger-directed aggression with regard to the sex of the stranger.

Although unanticipated and descriptive, our data from the undersocialized Chihuahuas and Chihuahua mixes revealed that most were assessed as showing concerning or dangerous behavior during the Stranger test, yet all were adopted, and the likelihood of return for them was similar to that previously recorded for all small dogs at our study shelter over a 5-year period [30]. These data from 26 undersocialized small dogs from a single home, together with data from a much larger number of dogs of all sizes displaying moderate to extreme fear in the shelter environment [28], show that positive outcomes for fearful dogs are not only possible but likely when given time and behavioral support.

## Figures and Tables

**Table 1:** Demographic characteristics of shelter dogs in relation to whether they were tested with either a male stranger (*n* = 55) or a female stranger (*n* = 202) during the Stranger test of the canine behavioral evaluation.

Demographic Characteristics of Dogs	Tested with Male Stranger	Tested with Female Stranger
Sex		
Male	41.8% (23/55)	43.6% (88/202)
Female	58.2% (32/55)	56.4% (114/202)
Reproductive status		
Intact	47.3% (26/55)	53.5% (108/202)
Neutered/spayed	52.7% (29/55)	46.5% (94/202)
Age class ^1^		
Juvenile	25.5% (14/55)	19.8% (40/202)
Adult	63.6% (35/55)	71.8% (145/202)
Senior	10.9% (6/55)	8.4% (17/202)

^1^ Juveniles, from 4 months to <1 year; adults, from 1 year to <8 years; and seniors, ≥8 years.

**Table 2:** Score on the Stranger test of the canine behavioral evaluation in relation to the sex of the stranger and demographic characteristics of shelter dogs (*n* = 257). Scores ranged from 1 (calm and friendly) to 5 (alarm barks, hackles up, growls, cannot settle, and will not approach the stranger upon solicitation). Sample sizes of dogs are in parentheses. Within specific variables, values with different superscript letters are significantly different (*p* < 0.05).

Variables	Score on the Stranger Test (Mean ± *SD*)
Sex of stranger	
Male	2.22 ± 1.29 (55) ^a^
Female	1.81 ± 1.09 (202) ^b^
Sex of dog	
Male	1.92 ± 1.14 (111)
Female	1.89 ± 1.15 (146)
Reproductive status of dog	
Intact	2.06 ± 1.21 (134) ^a^
Neutered/spayed	1.73 ± 1.05 (123) ^b^
Age class of dog ^1^	
Juvenile	1.80 ± 1.12 (54)
Adult	1.98 ± 1.18 (180)
Senior	1.52 ± 0.79 (23)

^1^ Juveniles, from 4 months to <1 year; adults, from 1 year to <8 years; and seniors, ≥8 years.

## Data Availability

All data from this study are included in Supplementary Materials.

## Supplementary Materials

The following are available online at https://www.mdpi.com/article/10.3390/ani13152461/s1, File S1 McGuire and Song Stranger Test Data.

## Appendix A

Table A1 provides brief descriptions of the tests and subtests of the canine behavioral evaluation at our study shelter. Details of the Stranger test are presented in Section 2.3.

**Table A1:** Tests and subtests of the Tompkins County SPCA’s canine behavioral evaluation.

Test	Subtest	Evaluator’s Actions
Cage presentation	Confrontational	Faces dog, bends at the waist, and makes direct eye contact
	Friendly	Faces sideways, bends down, and speaks to the dog in a friendly manner
Sociability	Stand and ignore	Stands and ignores the dog for 60 s
	Stroke three times	Slowly strokes dog from neck to tail three times
	Sit and ignore	Sits and ignores dog for 5 s
	Pet and talk	Sits, pets, and talks to the dog for 20 s in a friendly manner
Teeth exam		Makes five attempts to lift the dog’s upper lip and hold for 5 s
Handling	Far side	Stands perpendicular to the dog and strokes its far side
	Hind foot	Runs hand down the dog’s back and picks up hind foot
	Tail tug	Runs hand down the dog’s tail and tugs slightly
	Check ears	Touches and looks inside both ears
	Press shoulders	Applies slight pressure to the dog’s shoulders
	Lead by collar	Moves the dog around by collar
	Wipe with towel	Wipes the dog’s body with a towel
	Hug	Gives the dog a hug
Arousal		Initiates play with the dog with toys for 30 s and stops
Food bowl		Gives the dog a mix of kibble and canned food in a bowl, and using the Assess-a-Hand, strokes the dog’s back and attempts to pull the bowl away
Possession		Gives the dog a raw hide chew or pig’s ear, and using the Assess-a-Hand, attempts to take the item away
Stranger		See Section 2.3
Dog-to-dog ^1^	See a dog	Holds leash of the dog being tested while the scribe brings into the conference room a leashed, unfamiliar dog from the adoption floor
	Meet a dog	Allows dogs to interact while leashed

^1^ Previously tested dogs that did not show aggression toward other dogs during their own evaluation serve as stimulus dogs in this test.
